# SFE-CO_2_ Extract from *Typhonium giganteum* Engl. Tubers*,* Induces Apoptosis in Human Hepatoma SMMC-7721 Cells Involvement of a ROS-Mediated Mitochondrial Pathway

**DOI:** 10.3390/molecules16108228

**Published:** 2011-09-28

**Authors:** Qingyong Li, Chunfei Jiang, Yuangang Zu, Zhen Song, Baoyou Zhang, Xiangdong Meng, Wei Qiu, Li Zhang

**Affiliations:** 1 Key Laboratory of Forest Plant Ecology, Ministry of Education, Northeast Forestry University, Harbin 150040, China; Email: jiangchunfei2009@126.com (C.J.); 2 Engineering Research Center of Forest Bio-preparation, Ministry of Education, Northeast Forestry University, Harbin 150040, China; Email: zygorl@vip.hl.cn (Y.Z.); songzhen55@163.com (Z.S.); 605764896@qq.com (B.Z.); 289122789@qq.com (X.M.); qiuwei3042@126.com (W.Q.); ally521@126.com (L.Z.)

**Keywords:** *Typhoniumgiganteum* Engl., SFE-CO_2_ extract, SMMC-7721, Apoptosis, ROS

## Abstract

*Typhonium giganteum* Engl. (BaiFuzi) is one of the herbs commonly used in traditional Chinese medicine against cancer. In our previous studies, 37 compounds were identified the SFE-CO_2_ (supercritical fluid extraction with CO_2_) extract by GC-MS, including the four major components [β-sitosterol (40.22%), campesterol (18.45%), *n*-hexadecanoic acid (9.52%) and (*Z,Z*)-9,12-octadecadienoic acid (8.15%)]. The anti-cancer mechanisms of the SFE-CO_2_ extract from *T. giganteum* Engl. tubers have not been reported as yet. In this paper, the molecular mechanisms of the SFE-CO_2_ extract-mediated apoptosis in SMMC-7721 cells were further examined. SFE-CO_2_ extract inhibited the growth of SMMC-7721 cells in a time- and dose-dependent manner, arrested the cell cycle in the S phase and G2/M phase, and induced apoptosis. In addition, reactive oxygen species (ROS) increase, reduction of mitochondrial membrane potential, a rise in intracellular calcium levels were found in SMMC-7721 cells after treated with the extract. Western blot analysis showed that the extract caused down-regulation of Bcl-2 expression, and up-regulation of Bax expression. Moreover, caspase-3 and caspase-9 protease activity significantly increased in a dose-dependent manner. Collectively, our results showed that the SFE-CO_2_ extract from *T. giganteum* Engl. tubers induces apoptosis in SMMC-7721 cells involving a ROS-mediated mitochondrial signalling pathway.

## 1. Introduction

Apoptosis is a form of programmed cell death which occurs through activation of the cell-intrinsic suicide machinery [1] and is a hallmark of the action of many anticancer drugs [2,3,4]. Mitochondria play a pivotal role during the process of cell apoptosis which involves in a variety of key events, including loss of mitochondrial membrane potential (MMP), mitochondrial swelling and release of apoptotic proteins [5]. Activation of the apoptotic cascade results from a complex interaction of molecular events [6]. ROS, a group of highly reactive molecules, including singlet oxygen, hydroxyl radicals, superoxide anion, nitric oxide and hydrogen peroxides, have been shown to play a key role in apoptotic cell death [7]. ROS are known to induce the collapse of MMP, therefore trigger a series of mitochondria-associated events including apoptosis [8]. Excessive ROS generation can induce redox-signaling pathways, including oxidative stress, a rise in intracellular calcium levels, loss of cell function, cell cycle arrest, and apoptosis [9,10]. The mitochondria-dependent pathway for apoptosis is governed by Bcl-2-family proteins [11]. Bax/Bcl-2 regulates caspase-9 and caspase-3, which eventually leads to apoptosis [12].

The dried tuber of *Typhonium giganteum* Engl. is recorded in the Chinese Pharmacopoeia as a traditional Chinese medicine named Baifuzi [13]. The tuber of *T. giganteum* Engl. has been effectively used in Chinese folk medicine to treat cerebral apoplexy, dispel wind-phlegm, tumor-related diseases and many other illnesses [14]. It has been reported that the chemical components of *T. giganteum* Engl. tubers included β-sitosterol, β-sitosterol-D-glucoside, dl-inositol, cerebroside, *etc*. [15,16]. It also has been reported that the chemical constituents of the volatile oils from *T. giganteum* Engl. tubers included N-phenylbenzenamine, 2,6,10,14-tetramethylhexadecane, 6-methyl-2-phenylquinoline, *etc.* [17]. Several studies have reported that *T. giganteum* Engl. had potent anticancer activity, both *in vitro* and *in vivo* [18,19,20,21]. The aqueous extract from *T. giganteum* Engl. tubers induced apoptosis in SMMC-7721 cells via cell cycle arrest in S phase. The aqueous extract induces apoptosis in MCF-7 cells via cell cycle arrest in S and G2/M phase [19,20]. However, the chemical composition of the aqueous extract has not been revealed. In our previous studies, 37 compounds were identified in the SFE-CO_2_ extract by GC-MS, including the four major components [β-sitosterol (40.22%), campesterol (18.45%), *n*-hexadecanoic acid (9.52%) and (*Z,Z*)-9,12-octadecadienoic acid (8.15%)] [22]. In this paper, we explored the mechanisms of the SFE-CO_2_ extract from *T. giganteum* Engl. tubers-mediated apoptosis in human hepatoma SMMC-7721 cells. The results of this investigation might provide a scientific explanation for the traditional application of this herbal medicine in hepatic cancer therapy.

## 2. Results and Discussion

### 2.1. Cytotoxicity Assay

**Figure 1:**
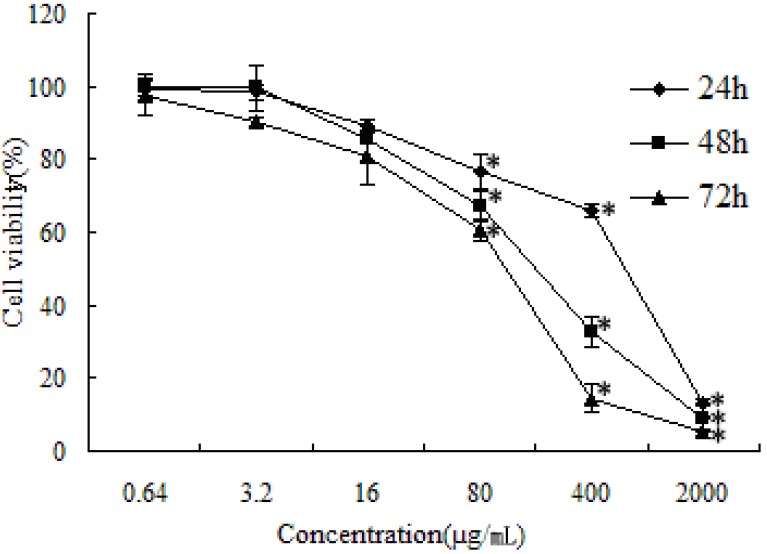
Effect of SFE-CO_2_ extract towards SMMC-7721 cells as determined by the MTT assay. The values are representative of three separate experiments, data are presented as mean ± S.D. * p < 0.05; p value compared with the control group.

Cell viability was determined by the MTT assay. In our previous studies, SFE-CO_2_ extract revealed different cytotoxic activities towards the seven human cancer cell lines (SMMC-7721, SGC-7901, HO-8910, A549, PC-3, MCF-7 and HCT-8). SMMC-7721 cells were the most sensitive cell line, thus, it was selected as a representative cell line for further investigation [22]. As shown in Figure 1, the growth of SMMC-7721 cells was significantly inhibited in a dose- and time-dependent manner by increasing concentrations of the extract after 24, 48 and 72 h. When SMMC-7721 cells were treated with 400 μg/mL SFE-CO_2_ extract, 67.29 ± 5.48% of cells were killed after 48 h.

### 2.2. Analysis of Cell Cycle Distribution

**Figure 2:**
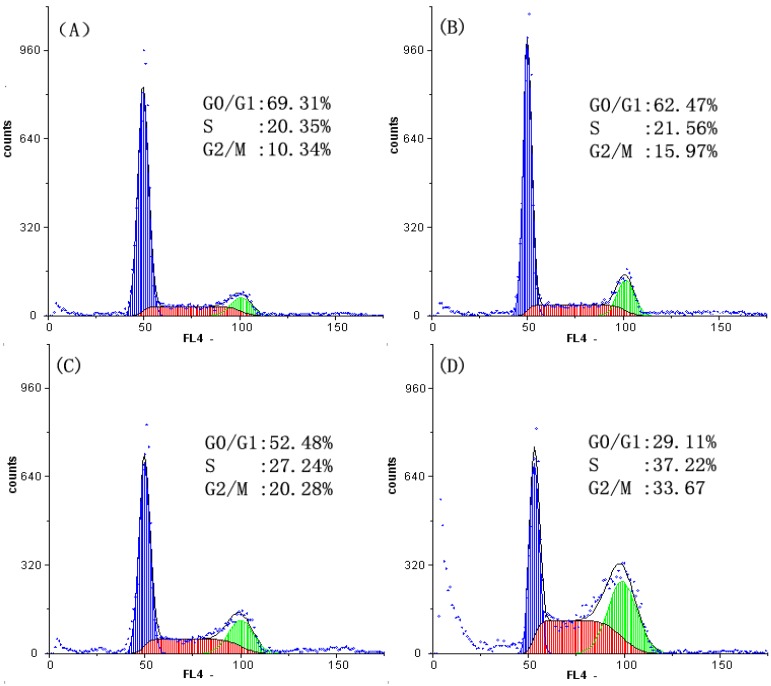
Effect of SFE-CO_2_ extract on the cycle of SMMC-7721 cells assayed by flow cytometry analysis of DNA content. (**A**) Untreated control cells; (**B**)-(**D**) treatment with 50, 100 and 150μg/mL SFE-CO_2_ extract.

The cells were treated with SFE-CO_2_ extract for 48 h, and the level of cell cycle progression was monitored by flow cytometry. As shown in Figure 2, a significantly increase in S phase populations was found after treated with SFE-CO_2_ extract, compared with control cells (from 20.35 ± 2.11% to 37.22 ± 2.53%). Meanwhile, a significant increase in G2/M phase was also found (from 10.34 ± 2.26% to 33.67 ± 2.38%). Hence, SFE-CO_2_ extract exerted growth-inhibitory effects via S phase and G2/M phase arresting in a concentration-dependent manner. The aqueous extract from *Typhonium giganteum* Engl. tubers arrested the cell cycle in the S phase in SMMC-7721 cells [19], the different ingredients between the aqueous extract and the SFE-CO_2_ extract showed different growth-inhibitory effects.

### 2.3. Cell Apoptosis Analysis

To further confirm the apoptosis induced by SFE-CO_2_ extract, AnnexinV-FITC/PI staining assay was used.

**Figure 3:**
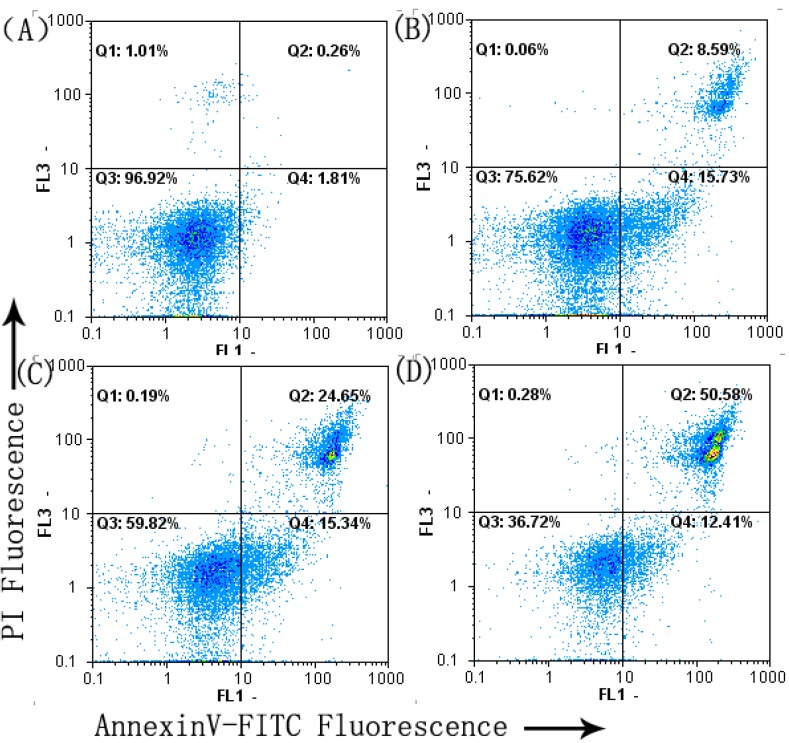
Effect of SFE-CO_2_ extract on the apoptosis of SMMC-7721 cells assayed by flow cytometry analysis of Annexin V-FITC/PI double stained cells. (**A**) Untreated control cells; (**B**)-(**D**) treatment with 50, 100 and 200 μg/mL SFE-CO_2_ extract.

The results showed that after treatment with SFE-CO_2_ extract for 48 h, the early and median apoptotic cells (Q4) were increased (1.81 ± 0.24% into 15.73 ± 2.24%, 15.34 ± 2.05% and 12.41 ± 1.97%, respectively) and the late apoptotic and necrotic cells (Q2) were increased (0.26 ± 0.02% into 8.59 ± 1.13%, 24.65 ± 2.68% and 50.58 ± 3.16%, respectively, Figure 3). These results suggested that SFE-CO_2_ extract was able to induce apoptosis of SMMC-7721 cells in a dose-dependent manner.

### 2.4. Changes in Nuclear Morphology

**Figure 4:**
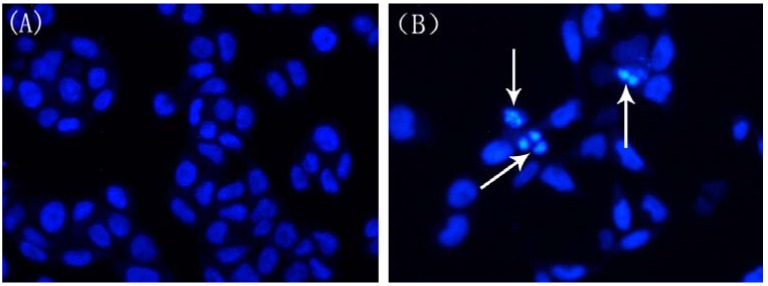
Morphological observation of SMMC-7721 cells treated with SFE-CO_2_ extract for 48 h by fluorescence microscopy. Cells undergoing apoptosis and nuclear fragmentation are indicated by arrows. (**A**) Untreated control cells; (**B**) treatment with 200 μg/mL SFE-CO_2_ extract. The representative images of only 2 pictures independent experiments are shown. Magnification: 200×.

To further investigate whether the SFE-CO_2_ extract mediated cell death in SMMC-7721 cells due to an apoptotic mechanism, the morphological changes were observed under inverted fluorescence microscope by Hoechst 33258 staining. Figure 4 shows that the nuclei of untreated control SMMC-7721 cells were stained in less bright blue and homogeneous color, but the cells treated with the 200 μg/mL extract for 48 h displayed typical apoptotic features including chromatin condensation and nuclear fragmentation [23]. White arrows pointed at the condensed chromatin. All of these changes suggested that the extract could induce apoptosis toward SMMC-7721 cells.

### 2.5. SFE-CO_2_ Extract Decreases Mitochondrial Membrane Potential( MMP) in SMMC-7721 Cells

**Figure 5:**
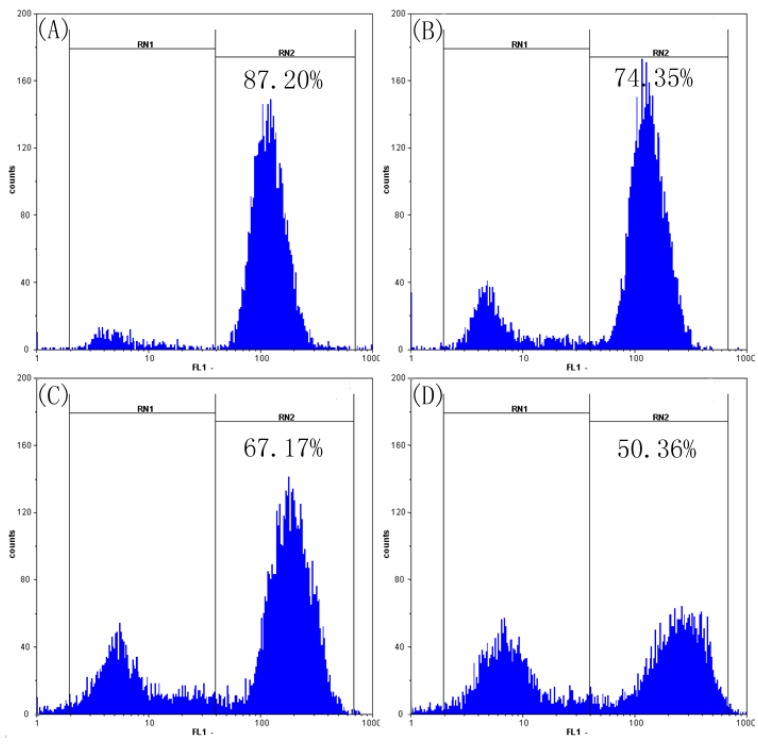
Effects of SFE-CO_2_ extract on MMP of SMMC-7721 cells assayed by flow cytometry analysis of Rh 123 stained cells. (**A**) Untreated control cells; (**B**)-(**D**) treatment with 50, 100 and 200 μg/mL SFE-CO_2_ extract.

The disruption of mitochondrial integrity is one of the early events leading to apoptosis. Loss of MMP is an important event during the mitochondrial pathway of apoptosis [24,25,26], so we investigated whether SFE-CO_2_ extract could induce the loss of MMP in SMMC-7721 cells. As shown in Figure 5, the MMP decreased to 74.35% ± 3.01, 67.17 ± 3.28% and 50.36 ± 2.79% in cells treated with the extract at 50, 100 and 200 μg/mL, respectively. These results demonstrated that SFE-CO_2_ extract induced mitochondria damage and diminished MMP in SMMC-7721 cells in a concentration-dependent manner.

### 2.6. Effect of SFE-CO_2_ Extract on Intracellular ROS in SMMC-7721 Cells

Since a loss of MMP is associated with the generation of ROS [27], we detected the level of ROS in SMMC-7721 cells treated with various concentrations of SFE-CO_2_ extract for 48 h with the cellular oxidation of H_2_DCFDA, a probe that is oxidized to green fluorescent DCF by various peroxide-like ROS and nitroxide-derived reactive intermediates [28].

**Figure 6:**
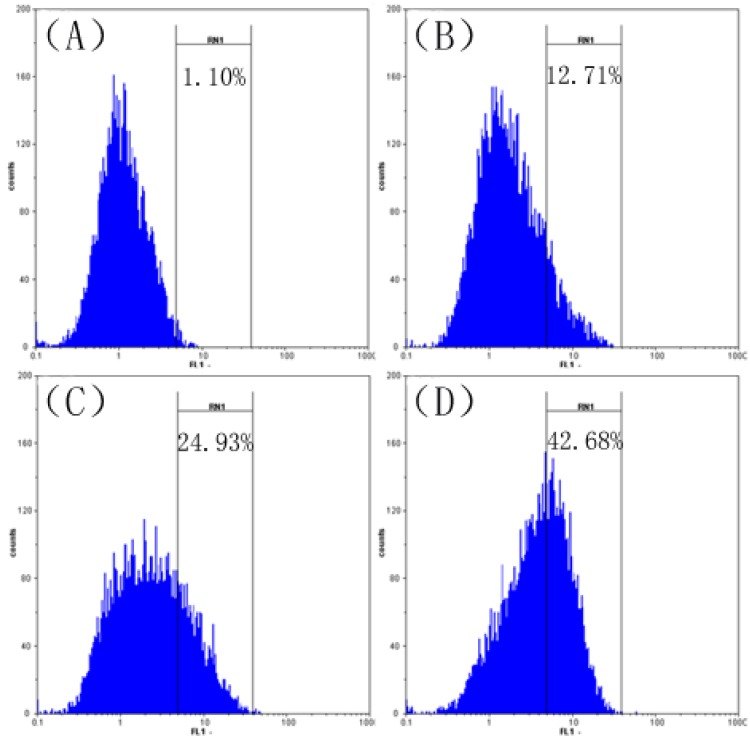
Effect of SFE-CO_2_ extract on ROS of SMMC-7721 cells assayed by flow cytometry analysis of DCFHDA stained cells. (**A**) Untreated control cells; (**B**)-(**D**) treatment with 50, 100, and 200 μg/mL SFE-CO_2_ extract.

As shown in Figure 6, the level of ROS in cells treated with SFE-CO_2_ extract was increased in a concentration-dependent manner, the level of ROS fluorescence increased to 12.71 ± 1.45%, 24.93 ± 3.64% and 42.68 ± 3.22% in cells treated with SFE-CO_2_ extract at 50, 100 and 200 μg/mL, respectively. These data demonstrated that SFE-CO_2_ extract significantly increased ROS production in SMMC-7721 cells. ROS production may promote mitochondrial dysfunction and trigger mitochondria-mediated apoptosis.

### 2.7. Effect of SFE-CO_2_ Extract on Intracellular Calcium Concentration ([Ca^2+^]_c_) in SMMC-7721 Cells

**Figure 7:**
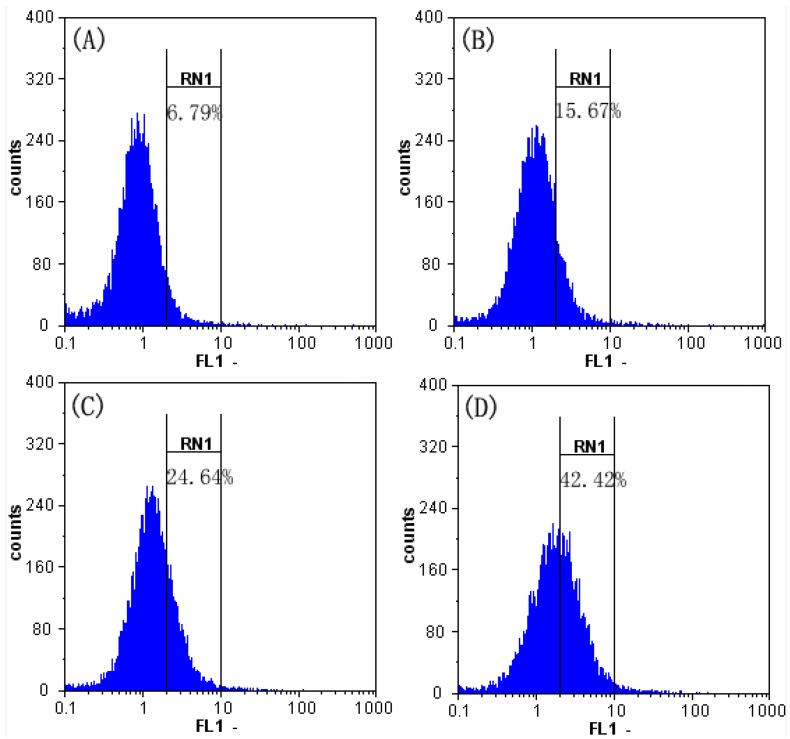
Effect of SFE-CO_2_ extract on intracellular calcium levels of SMMC-7721 cells assayed by flow cytometry analysis of Fluo-3/AM stained cells. (**A**) Untreated control cells; (**B**)-(**D**) treatment with 50, 100and 200 μg/mL SFE-CO_2_ extract.

Some studies reveal that ROS inactivates some transporters, which lead to a rise in [Ca^2+^]_c_ and subsequent cell dysfunction [10]. We determined the intracellular Ca^2+^ concentration using flow cytometry. The fluorescence intensities of the control and treated groups were not coincident, and the intensity of the treated group was shifted to the right [29]. The [Ca^2+^]_c_ increased to 15.67 ± 2.01%, 24.64 ± 2.75% and 42.42 ± 3.81% in cells treated with the extract at 50, 100 and 200 μg/mL, respectively (Figure 7). The results indicated that SFE-CO_2_ extract-induced [Ca^2+^]_c_ increase in a dose-dependent manner in SMMC-7721 cells.

### 2.8. Activation of Caspase-3 and -9 by SFE-CO_2_ Extract

**Figure 8:**
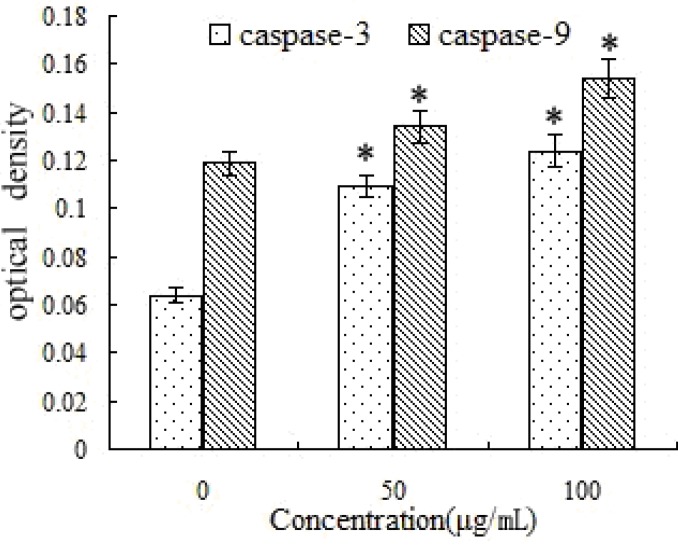
Effects of SFE-CO_2_ extract for regulating caspase-9 and caspase-3 in SMMC-7721 cells. The results are expressed as the mean ± S.D. of three independent experiments. *p < 0.05, p value compared with the control group.

Caspases, which are a family of cysteine proteases, play key roles in executing the apoptotic process. Once activated, caspases activate downstream caspases, leading to apoptosis [30,31]. In the untreated SMMC-7721 cells, OD value of caspase-3 was 0.064 ± 0.007, and the OD value of caspase-9 was 0.119 ± 0.005. After treatment with SFE-CO_2_ extract, caspase-3 and -9 protease activity significantly increased in a dose-dependent manner. The highest activities of caspase-3 and caspase-9 were found upon exposure to 100 μg/mL SFE-CO_2_ extract. The OD values were 0.124 ± 0.012 and 0.154 ± 0.022, respectively, and were significantly higher than those in the control group (Figure 8).

### 2.9. SFE-CO_2_ Extract-Mediated Up-Regulation of Bax and Down-Regulation of Bcl-2

**Figure 9:**
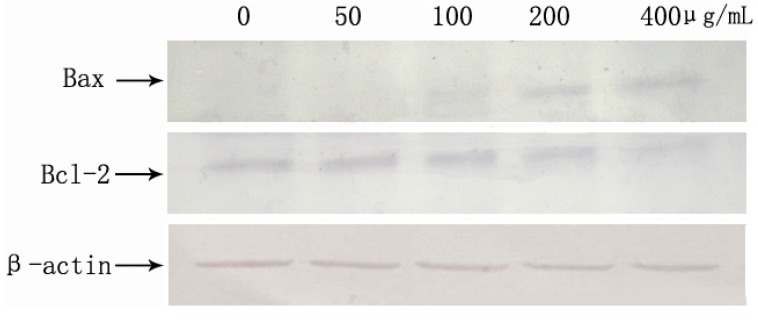
Effects of the SFE-CO_2_ extract on apoptosis-related proteins expression analyzed by Western blot.

Bcl-2 family proteins have a central role in controlling the mitochondrial pathway. The Bcl-2 family significantly regulates apoptosis either as an activator (e.g., Bax) or as an inhibitor (e.g., Bcl-2), Therefore, it has been suggested that the Bax/Bcl-2 ratio was a key factor in regulation of the apoptotic process [32,33]. We used Western blotting to measure the expression of the Bcl-2 family members. As shown in Figure 9, Western blot analyses revealed that the levels of pro-apoptotic Bax were significantly increased, whereas the levels of anti-apoptotic Bcl-2 were decreased in a concentration-dependent manner. These results indicate that apoptosis induced by SFE-CO_2_ extract is related to the mitochondrial pathway.

## 3. Experimental

### 3.1. Plant Material and Extraction

*T. giganteum* Engl. tubers were purchased from a commercial source (*Typhonium giganteum* Engl. Planting and Development Base Company, Jilin, China) and authenticated by Prof. Shao-Quan Nie (Key Laboratory of Forest Plant Ecology, Northeast Forestry University, Harbin, China). SFE-CO_2_ extract from *T. giganteum* Engl. tubers was prepared in our laboratory [22]. Briefly, the tubers were sliced and air-dried at room temperature to a final moisture content below 0.5%. Air-dried tubers were pulverized in a milling machine and then sieved (40 mesh). The SFE-CO_2_ extraction experiments were performed using an HA121-50-01 SFE device (Hua’an Supercritical Fluid Extraction Corp., Nantong, China). The extraction vessel pressure and temperature were 30 MPa and 45 °C, separation vessel pressure and temperature were 5 MPa and 50 °C, and CO_2_ flow rate of 15 kg·h^−1^. After the scheduled time, the extraction vessel was depressurized and the oil was collected from the separation vessel. The extract was concentrated on a vacuum rotary evaporator under reduced pressure, and stored at −20 °C.

### 3.2. Maintenance of Human Cancer Cell Lines

Human hepatoma cancer SMMC-7721 cell line was purchased from China Center for Type Culture Collection (Wuhan, China). All the cells were cultured in RPMI-1640 medium supplemented with 10% fetal bovine serum and 100 U/mL penicillin and 100 μg/L streptomycin in a humidified atmosphere of 5% CO_2_ at 37 °C.

### 3.3. Cytotoxicity Assay

The cytotoxic effects of the SFE-CO_2_ extract on SMMC-7721 cells were assayed by the 3-(4,5-dimethylthiazol-2-yl)-2,5-diphenyltetrazolium bromide (MTT) assay [34]. The cells were plated separately in 96-well culture plates (5 × 10^4^ cells/well). After 24 h incubation, cells were treated with SFE-CO_2_ extract (0, 0.64, 3.2, 16, 80, 400 and 2,000 μg/mL, five wells per concentration) for 24, 48 or 72 h, MTT solution (5 mg/mL) was then added to each well. After 4 h of incubation, the formazan precipitate was dissolved in DMSO (100 μL) and then the absorbance was measured in an ELISA reader (Thermo Molecular Devices Co., Union City, CA, USA) at 492 nm. The cell survival curves were calculated after comparing with the control. The percentage viability was calculated as follows:

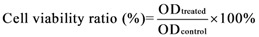



### 3.4. Cell Cycle Distribution

Cell cycle was assayed with CyStain (Partec GmbH, Görlitz, Germany) [35]. SMMC-7721 cells (1 × 10^6^ cells/well) were seeded in a 6-well plate for 24 h and then exposed to SFE-CO_2_ extract (0, 50, 100, 150 μg/mL). After incubation for 48 h, untreated and treated cells were collected and washed twice with precooled PBS, and then suspended in CyStain (200 μL) and PBS (800 μL). The cell cycle distribution of 10,000 cells was determined by flow cytometry (Partec GmbH, Germany) using the FloMax software (Partec GmbH).

### 3.5. Apoptosis Assays

Apoptosis assay was performed using an rh Annexin V-FITC Kit (Bender MedSystems GmbH, Vienna, Austria) as described in the manufacturer’s instructions. SMMC-7721 cells were cultured in medium containing different concentrations of SFE-CO_2_ extract (0, 50, 100, 200 μg/mL) for 48 h, collected and washed twice with PBS, gently resuspended in annexinV-FITC binding buffer (1×, 195 μL) and incubated with annexinV-FITC (5 μL) in the dark for 10 min at 25 °C. The cells were then centrifuged at 3,000 rpm for 5 min, gently resuspended in annexinV-FITC binding buffer (1×, 190 μL) and PI (10 μL) was added, followed by immediate analysis by flow cytometry.

### 3.6. Nuclear Staining with Hoechst33258

Morphological observation of nuclear change was assayed with Hoechst 33258 [36]. SMMC-7721 (1 × 10^6^ cells/mL) cells were seeded in 6-well plates. After adherence for 24 h, the cells were cultured in medium containing different concentrations of SFE-CO_2_ extract (0, 50, 100, 200 μg/mL) for 48 h at 37 °C. The cells were washed twice with cold PBS and fixed with 4% paraformaldehyde in PBS for 30 min, stained with Hoechst 33258 (10 μg/mL) for 10 min at 37 °C, and then subjected to fluorescence microscopy (Nikon ECLIPSE TE2000-E, Tokyo, Japan).

### 3.7. Measurement of ROS Generation

ROS generation was monitored by flow cytometry using DCFHDA [28]. Briefly, SMMC-7721 cells (1 × 10^6^ cells/well) were seeded in a 6-well plate for 24 h and then exposed to SFE-CO_2_ extract (0, 50, 100, 200 μg/mL). After incubation for 48 h, cells were collected and suspended with DCFH-DA (10 μM) at 37 °C for 30 min. Fluorescence generation was due to the hydrolysis of DCFHDA to dichlorodihydrofluorescein (DCFH) by non-specific cellular esterases, and the subsequent oxidation of DCFH by peroxides was measured by means of flow cytometry.

### 3.8. The Changes of Mitochondrial Membrane Potential (MMP)

Mitochondrial membrane potential was measured by flow cytometry with rhodamine 123 [37,38]. SMMC-7721 cells (1 × 10^6^ cells/well) were seeded in a 6-well plate. After adherence for 24 h, cells were treated with serial dilutions of SFE-CO_2_ extract (0, 50, 100, 200 μg/mL) for 48 h. Cells were harvested and incubated with rhodamine 123 (10 μg/mL) at 37 °C for 30 min, followed by immediate analysis by flow cytometry at 488 nm.

### 3.9. Intracellular Calcium Analysis

The concentration of calcium was measured using Ca^2+^ indicator Fluo-3/AM as described previously [39]. SMMC-7721 cells (1 × 10^6^ cells/well) were seeded in a 6-well plate. After adherence for 24 h, the cells were treated with different concentrations of SFE-CO_2_ extract (0, 50, 100, 200 μg/mL) for 48 h. Fluo-3/AM (Sigma, 5 μM) was added to the treated cells for 30 min at 37 °C. The cells were then analyzed immediately by flow cytometry.

### 3.10. Measurement of Caspase-3 and Caspase-9 Activities

Activity of caspase-3 and caspase-9 were determined with a colorimetric kit (Nanjing kaiji Bio-Tek Corporation, Nanjing, China) [40]. SMMC-7721 cells were treated with SFE-CO_2_ extract (0, 50, 100 μg/mL) for 48 h, respectively. The cells (1 × 10^6^ cells/mL) were harvested and washed once with PBS. After the SMMC-7721 cells were lysed, reaction buffer was added to the SMMC-7721 cells followed by the additional 5 μL of caspase-3 or caspase-9 colorimetric substrate (DEVD-pNA) and incubated in a 96-well plate for 4 h at 37 °C in a CO_2_ incubator. The plate was then measure with an ELISA reader at an absorbance of 405 nm. Activities of caspase-3 and caspase-9 were expressed relative to theoretical density value (OD).

### 3.11. Western Blot Analysis

SMMC-7721 cells were treated with SFE-CO_2_ extract (0, 50, 100, 200, 400 μg/mL) for 48 h, respectively. For isolation of total protein fractions, cells were collected, washed twice with ice-cold PBS, and lysed using cell lysis buffer [20mM Tris pH 7.5, 150mM NaCl, 1% Triton X-100, 2.5 mM sodium pyrophosphate, 1 mM EDTA, 1% Na_3_CO_4_, 0.5 μg/mL leupeptin, 1 mM phenylmethane-sulfonyl fluoride (PMSF)]. Equal amounts of lysate protein were run on 12% SDS–PAGE and electrophoretically transferred to PVDF membrane. After blocking, the blots were incubated with specific primary antibodies (anti-Bcl-2 and anti-Bax antibodies) overnight at 4 °C and further incubated for 1 h with an alkaline phosphatase peroxidase-conjugated respective secondary antibody. Detection was performed by the BCIP/NBT Alkaline Phosphatase Color Development Kit (Beyotime Institute of Biotechnology, Jiangsu, China) according to the manufacturer’s instructions. Bands were recorded by a digital camera (Canon, EOS 350D, Tokyo, Japan).

### 3.12. Statistical Analysis

Data are expressed as the mean ± SD. Statistical analysis of group differences was performed using Student’s t-test. A value of p < 0.05 was considered statistically significant.

## 4. Conclusions

In summary, the present study showed that SFE-CO_2_ extract from *T. giganteum* Engl. tubers could induce ROS production, lead to loss of MMP, increase the ratios of Bax/Bcl-2, activate stress-responsive caspase-9 and caspase-3 in SMMC-7721 cells. These results suggested that SFE-CO_2_ extract induced apoptosis involving a ROS-mediated mitochondrial pathway in SMMC-7721 cells. *T. blumei* and *T. flagelliforme* are other *Typhonium* species. It has been reported that *T. blumei* induced A549 cells apoptosis via the mitochondrial pathway [41]. *T. flagelliforme* induced CEMss cells apoptosis via the mitochondrial pathway [42]. These dates were consistent with the results of our study. The SFE-CO_2_ extract included the four major components (β-sitosterol, campesterol, n-hexadecanoic acid and (*Z,Z*)-)9,12-octadecadienoic acid [22]. We presume that the four major compounds play a major role in apoptosis in SMMC-7721 cells. Further studies are in progress about activity of SFE-CO_2_ extract towards SMMC-7721 xenograft tumors in nude mice.
